# Nigeria’s public health response to disease outbreaks: A review of strengths and weaknesses

**DOI:** 10.4102/jphia.v15i1.773

**Published:** 2024-12-18

**Authors:** Japhet H. Jonah, Gabriel Samuel, Mohammed I. Abdullahi, Edwin A. Emmanuel, Samuel S. Danladi, Celestine E. Ekwuluo

**Affiliations:** 1 Department of Nursing, FHI360, Maiduguri, Nigeria; 2 International Humanitarian Action - (NOHA) - Institute for International Law of Peace and Armed Conflict (IFHV), Ruhr Universität, Bochum, Germany; 3 Department of Public Health, Liverpool John Mores University, Liverpool, United Kingdom; 4 Health and Nutrition Emergency Response Team, Mediar, Juba, Sudan; 5 Department of Public Health, Ahmadu Bello University, Zaria, Nigeria; 6 Department of Health, International Medical Corps (IMC), Kyiv, Ukraine

**Keywords:** infectious disease, outbreak, response, Nigeria, public health, strategy

## Abstract

**Background:**

In the past 20 years, Nigeria has confronted an array of six significant infectious disease outbreaks, including coronavirus disease 2019 (COVID-19), Lassa fever, meningitis, diphtheria, cholera and Ebola. Although the country has generally managed to address most of these infections effectively, there are still some shortcomings in the nation’s public health response.

**Aim:**

This review examined the six most significant outbreaks of infectious diseases and the corresponding public health responses, evaluating the strengths and limitations of the country’s public health system.

**Setting:**

This study focused on Nigeria. Nigeria is regarded as a country with the largest population in Africa.

**Method:**

A narrative review approach was employed, which entailed identifying pertinent literature using search terms with various Boolean combinations derived from multiple electronic databases, including PubMed, Google Scholar, the National Institutes of Health (NIH) database, the Web of Science and Africa Journals Online.

**Results:**

The key strengths of Nigeria’s public health system include surveillance, workforce development, prevention at entry points, risk communication and establishment of national reference libraries. However, the study also identified several areas of weakness, such as inadequate funding, inadequate efforts at the subnational level, poor coordination between public health and security authorities, a lack of integration between the animal and human health sectors, inadequate biosafety and biosecurity policies and programmes and logistical complexities.

**Conclusion:**

Despite the strengths, there are still weaknesses within Nigeria’s public health system response to infectious diseases.

**Contribution:**

The study outlined the various strengths and weaknesses of the Nigeria Public health system in combating infectious disease. Therefore, it is recommended that funding be increased, poverty and inequalities are addressed, efforts at the subnational level be increased, and a Memorandum of Understanding (MOU) between the public health sector and security authorities be established.

## Introduction

Nigeria is regarded as the most populous country in Africa and the sixth most populous globally, with over 200 million people making up 15% of the African population.^1^ Arguably, high population growth could lead to economic growth. However, the reverse is the case for Nigeria, as population growth puts pressure on the available resources, leading to low productivity and standard of living, as explained by the Malthusian theory.^2^ This factor has led to decreased economic growth rates and a flattening gross domestic product (GDP) per capita. Additionally, poor monetary and unexpected external factors such as the coronavirus disease 2019 (COVID-19) pandemic have further exacerbated the situation, with more than 50% of the population living in multidimensional poverty.^3,4^

Notably, poverty is a significant risk factor for contracting infectious diseases, as impoverished living conditions heighten susceptibility and exposure. Moreover, the disease outcomes for individuals experiencing multidimensional poverty often prove to be more adverse because of inadequate access to quality healthcare, poor underlying conditions, co-morbidities and the absence of social support.^5^ Consequently, the current economic crisis in Nigeria has put the nation’s already vulnerable public health system in an even more precarious position, not just because of untrained personnel but also because of insufficient government funding, mismanagement of scarce resources and a rising cost of living.^2^

Despite the challenges and weak public health system, Nigeria was able to overcome several deadly infectious diseases such as COVID-19, Lassa fever (LF) and Ebola.^6^ Therefore, this article seeks to review how the Nigerian public health system responded to major infectious diseases in the past two decades (2002–2022). Reviewing responses to previous outbreaks in the recent past is necessary to create a comprehensive national outbreak preparedness and response plan. Additionally, a narrative review of Nigeria’s public health response across various health challenges will provide insights into areas of strength and potential improvement that will aid appropriate policy changes. Finally, understanding the lessons learnt can inform Nigeria and other countries on effective strategies and pitfalls to avoid future public health crises.

### Aim and objectives

The general aim of this narrative review is to assess Nigeria’s public health response to major infectious disease outbreaks in terms of severity (COVID-19, LF, meningitis, diphtheria, cholera and Ebola) from 2002 to 2022. This will be achieved via the following specific objective:

- To discuss the strengths and weaknesses of Nigeria’s Public Health Response to the major infectious diseases (COVID-19, LF, meningitis, diphtheria, cholera and Ebola) using the 2023 Joint External Evaluation (JEE).

## Methods

A narrative literature review approach was adopted to provide an exhaustive overview of the existing literature on the strengths and weaknesses of public health response to six major infectious disease outbreaks in Nigeria.

### Sources of information

The literature search was performed between January 2024 and July 2024 using several electronic databases, websites and organisations to identify pertinent studies on public health responses to outbreaks of infectious diseases in Nigeria. The searched databases include PubMed, Google Scholar, the National Institutes of Health database, Web of Science and Africa Journals Online. The organisational websites comprise the World Health Organization (WHO), the Nigeria Centre for Disease Control (NCDC) and World Bank websites.

### Literature search

Relevant articles were identified using terms such as ‘Nigeria’, ‘outbreak’, ‘public health’, ‘infectious disease’, ‘response’ and ‘strategies’ in differing Nesting and Boolean combinations. Keywords were extracted using the United States National Library of Medicine MeSH on demand. The publication date range was restricted to 2014–2024 to gather evidence from the past decade.

### Inclusion and exclusion criteria

Precise inclusion and exclusion criteria were developed before starting the literature search. Articles were included based on their relevance and contribution to the narrative review. The inclusion criteria stipulated that eligible studies must be original research published in English and focused on response strategies and interventions implemented for infectious disease outbreaks in Nigeria. Exclusion criteria eliminated opinion pieces, commentaries, editorials and articles that concentrated exclusively on outbreak epidemiology without discussing public health responses in Nigeria.

These criteria ensured that selected articles met the objectives of evaluating evidence on Nigeria’s outbreak response measures.

### Analysis

**FIGURE 1:**
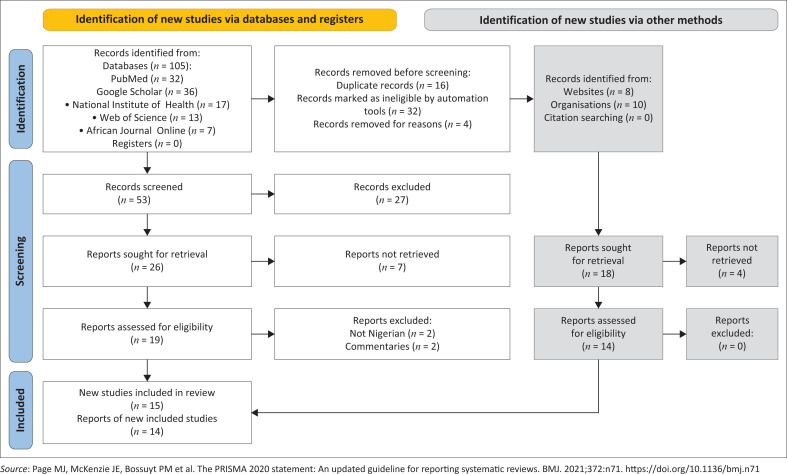
Flow diagram of the PRISMA illustrating the procedure for selecting studies.

After a search across all the specified databases, each article underwent a meticulous screening process using the Preferred Reporting Items for Systematic Reviews and Meta-Analyses (PRISMA) guideline. The process is illustrated in the PRISMA flow chart in Figure 1^7^, starting with a review of abstracts to check relevance and followed by a full-text review to confirm eligibility as per the predetermined criteria.

The results are presented in a narrative summary, accompanied by figures to illustrate key findings and concepts. The review is organised into eight main sections: (1) introduction to the topic, (2) methodology of the review, (3) overview of major infectious disease outbreaks and corresponding public health response, (4) strengths of Nigeria’s public health response, (5) weaknesses of Nigerian public health response, (6) recommendations on how to strengthen Nigeria’s response to infectious diseases and (8) conclusion.

### Ethical considerations

This article followed all ethical standards for research without direct contact with human or animal subjects.

## Results

### Major infectious disease outbreaks and the corresponding public health response in Nigeria from 2014 to 2024

From 2002 to 2022, Nigeria has reported five or more public health emergencies to the WHO annually.^7^ The most significant infectious disease outbreaks of public health importance in the past two decades include COVID-19, LF, Ebola, meningitis, diphtheria and cholera.^8^

**FIGURE 2:**
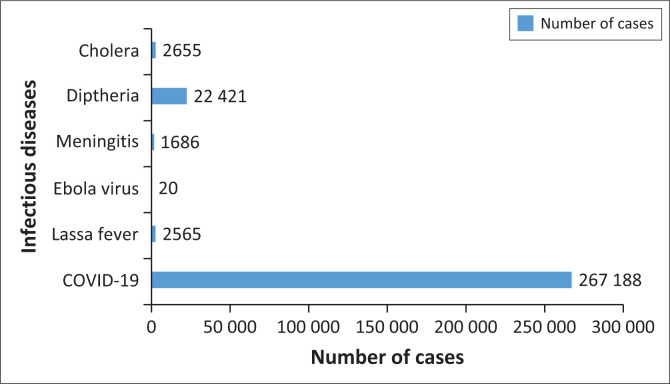
Number of cases of the major infectious diseases reported in Nigeria within different year periods.

From Figure 2, it can be observed that COVID-19 is responsible for most new cases from 2020 until 2024. On the other hand, the Ebola virus has the least number of new cases, amounting to 20, by the year 2023. However, it is essential to notice that Ebola has a higher case fatality rate of 40% compared to COVID-19’s 1.2%.^9,10^ In addition, diphtheria ranks second in new cases, with 22 421 cases reported. Nevertheless, compared to LF, which has had an incidence of 2565 from 2006 to 2022, LF’s case fatality rate of 7% is more than double that of diphtheria’s 3%.^11,12^ Therefore, it can be concluded that the six listed infectious diseases are of great importance to public health despite their varying incidence.

#### Coronavirus disease 2019

The index COVID-19 case in Nigeria was reported on 28 February 2020.^13^ As of 28 March 2021, Nigeria has reported over 160 000 cases of COVID-19 and about 2000 deaths because of the virus, making it the 77th most impacted nation in the world and the fifth in Africa. The attack rate was found to be 78.8 per 100 000 people, a death rate of 1.0 per 100 000 people and a 1.30 case fatality rate.^14^

In response to the COVID-19 pandemic, a stringent multifaceted approach involving the Presidential Task Force (PTF) was adopted. They were saddled with oversight functions, strategies and policy guidance. The Nigeria Center for Disease Control (NCDC) also worked with the PTF to ensure coordination, resource mobilisation, epidemiology and surveillance.^15^ The two bodies (PTF and NCDC) also worked with research institutes to ensure early response and non-pharmaceutical and clinical intervention. To ensure an early response, mobilisation and sanitisations, training of rapid response team (RRT), and emergency centres were set up. Non-pharmaceutical interventions include travel restrictions, curfew, contact tracing, self-isolation and quarantine. Furthermore, public health campaigns, hand hygiene, face masks and social distancing were ensured. On the other hand, clinical interventions include health training professionals, use of personal protective equipments (PPEs), isolation, case management and proper diagnostic testing.^4^

#### Lassa fever

Lassa fever is one of the most common recurrent infectious acute viral haemorrhagic fevers. It was first identified in Borno State of Nigeria in 1969.^16^ From 2009 to 2020, there were about 28 000 suspected cases, with about 1844 deaths in Nigeria.^12^

The public health response was undertaken by the Nigeria Centre for Disease Control and Prevention (NCDC) along with health officials of the impacted states. An NCDC Emergency Operations Center (EOC) was activated during the outbreak by deploying RRTs to the various states affected. Their response activities included surveillance, case management, prevention and control measures.^16^ To enhance the response to LF outbreaks, the NCDC created a national action plan in collaboration with WHO and other partners aimed at preventing and controlling these outbreaks. This strategy emphasised the enhancement of surveillance, the expansion and improvement of national laboratory capabilities, the raising of public awareness and the betterment of available case management.^16^

#### Ebola virus disease

The first index case of Ebola in Nigeria was found in 2014, imported by a traveller from Liberia.^17^ The disease quickly spread from this index case to a total of 19 laboratory-confirmed cases of Ebola virus disease (EVD), and one probable case was reported in two states. Out of the confirmed EVD cases in Nigeria, eight resulted in death, leading to a case fatality rate of 42.1%, while twelve individuals recovered fully.^18^

Three months after the first case was confirmed, WHO declared Nigeria to be Ebola-free by WHO, which was deemed a successful public health response. The swift management of the EVD was enabled by the quick identification of the initial case, thorough contact tracing efforts and the isolation and treatment of subsequent cases. Additionally, the success was attributed to the multi-sectorial approach, public–private partnerships and utilisation of faith-based organisations and the media. Furthermore, international community participation, handwashing facilities in public and private schools and social mobilisation using multimodal strategies also helped in the success.^18^

#### Meningitis

Between 01 October and 16 April 2023, 1686 potential cases have been reported, with 532 of those confirmed, leading to 124 fatalities case fatality rate (CFR: 7%). The NCDC has implemented response strategies at the national level with assistance from WHO. The response included case management, vaccination and surveillance activities.^19^ The mass vaccination campaigns and other response measures, such as better case management and case finding during the outbreak, helped control the epidemic.^19^ A significant turning point in managing the meningitis epidemics was the introduction of the meningococcal A conjugate vaccine against *Neisseria meningitidis* serogroup C (NmC).^20^

#### Diphtheria

Previous issues of diphtheria have been documented in Nigeria, with the worst case occurring in Borno State, in the northeast of the country, in 2011.^19^

In response to the outbreak, he NCDC collaborates with WHO and various partners to oversee public health initiatives, including vaccination responses, enhanced monitoring for prompt identification of cases, management of cases and communication of risks.^19^

#### Cholera

Cholera remains a significant public health concern in Nigeria, having persisted for approximately five decades. Although the outbreaks of this highly infectious illness are intermittent, it has been endemic in the country because the first epidemic case was reported in 1970.^21^ According to NCDC, over 3000 cases and 77 deaths were recorded in 2023 and 2024 alone.^22^

To control the cholera outbreak, the government, together with NCDC, implemented a community-based strategy that encompasses community engagement, coordination, immunisation, water, sanitation and Hygiene (WASH) programmes, disease surveillance and early detection, provision of oral rehydration salts, case management, laboratory services and risk communication which was proven to be effective.^23^

## Discussion

### The strengths of Nigeria’s public health response to infectious diseases

In recent years, Nigeria has made notable progress in enhancing its public health response to outbreaks of infectious diseases.^24^ The country has demonstrated the capacity to effectively manage infectious disease outbreaks, as exemplified during the Ebola crisis in 2014, which was controlled within 3 months.^18^ The strengths of the Nigeria public health response were analysed during the 2023 JEE.^25^

A JEE is an optional, collaborative and cross-cutting evaluation of the abilities of member countries to avert, identify and swiftly react to public health threats, such as infectious diseases. The JEE assists nations in identifying the most significant vulnerabilities in their healthcare systems to focus on areas for improved readiness and public health responses. It evaluates a nation’s ability to avert, identify and react to public health crises across 19 specific technical domains.^25^ The latest one was conducted in August 2023, which revealed a significant improvement in Nigeria’s public health response to infectious disease, with a notable rise from 39% in 2017 to 54% in 2023. The significant advancement is credited to the execution of the National Action Plan for Health Security (NAPHS) and the one-health strategy, which strengthened cooperation among different stakeholders and directed financial resources to rectify the recognised deficiencies in the public health system.^26^ The various areas of strength include the following:

#### Public health surveillance system

Nigeria’s public health surveillance system was significantly improved, notably the state-of-the-art Incident Control Centre (ICC) at the NCDC, collaborating with the Event Based Surveillance Unit (EBSU), as they collect and analysed data on disease outbreaks throughout the country, utilising several sources, such as online platforms, call centres, and rumour management tools known as the Epidemic Intelligence from Open Sources (EIOS). More so is involved the Surveillance Outbreak Response Management and Analysis System (SORMAS) for quick outbreak tracking and response and Early Warning, Alert and Response System (EWARS).^25^

Additionally, resources were allocated to establish disease surveillance systems, EOCs and mechanisms for coordinating response efforts. These combined measures have enhanced Nigeria’s capacity to promptly detect and respond to infectious disease outbreaks.^26^

#### National Reference Laboratory

The establishment of a National Reference Laboratory (NRL) that houses advanced technologies such as the Mega polymerase chain reaction (PCR) laboratory, Multiplex Bead Assay (MBA) Lab, Sequencing Lab, microbiology, serology and open system PCR lab suite has markedly improved the country’s public health response. The NRL also houses the National Biorepository Centre (NBC), accredited by the National Health Research Ethical Committee (NHREC), housing over a million samples from various national surveys for research, quality assurance and training purposes.^26^

#### Prevention at entry points

The country’s entry points were strengthened to prevent, detect and respond to infectious disease outbreaks. Competent authorities are stationed at six airports, five seaports, several ground crossings and trans-border markets.^27^ The WHO’s list of approved ports for ship inspections and health certificates now includes the eight Nigerian ports, which enhances Nigeria’s position in international maritime health security and facilitates thorough inspections of vessels for safety and biosecurity. Additionally, five international airports and several land border crossings implement inspection programmes to guarantee that the environment and facilities utilised by travellers are kept in clean and safe conditions.^16^

#### Strong risk communication and community engagement

Nigeria employed community engagement strategies to foster the adoption of preventive public health practices. The country collaborated with indigenous leaders and non-governmental entities to disseminate knowledge within communities regarding communicable diseases and strategies for their prevention.^26^

#### Workforce development

Over the review period, Nigeria has developed its workforce and maintained a well-trained public health workforce equipped with the necessary technical skills, scientific knowledge and subject-specific expertise. This contributed to strengthening the public health system.^27^ The Nigeria Field Epidemiology and Laboratory Training Programme (NFELTP) trained several hundred professionals, including epidemiologists, laboratorians and veterinarians.^16^

### Weaknesses of Nigeria’s public health response to infectious diseases

Despite the numerous strengths of Nigeria’s public health response, some areas still need improvement. The various weaknesses in the country’s public health system include the following:

#### Poor funding for preparedness

There is still a need for sustained and increased domestic financing for epidemic preparedness-related activities and gender equity in epidemic preparedness and response.^27^ The persistent lack of adequate funding for healthcare has resulted in deficiencies in essential equipment, supplies and personnel, particularly in rural regions. This phenomenon can impede the promptness of response efforts during disease outbreaks.^26^

#### A lack of animal sector integration

Significant advancements have been achieved in improving human health security in Nigeria. However, because of zoonotic infectious diseases, there is still a necessity to incorporate and advocate for the environmental and animal sectors within the wider health security framework to effectively enhance public health.^26^ The integration of these sectors is vital for a comprehensive one-health strategy that tackles the interconnections between human, animal and environmental health. Bridging this integration gap will enhance Nigeria’s capacity to predict and effectively respond to new health threats that frequently emerge at the interface of humans, animals and the environment.^26^

#### Poor efforts at the subnational level

Despite notable strides made at the national level, extending these advancements to the subnational regions, especially the local government areas is imperative. Strengthening capacity at this level ensures a more robust and decentralised response mechanism, enabling prompt actions in remote areas. Subnational capacity building entails providing adequate training, resources and infrastructure to local health authorities, ensuring that Nigeria’s health security improvements are felt throughout the country.^27^

#### Poor linkage between public health and security authorities

During a public health crisis, law enforcement must promptly work together with public health and medical authorities to coordinate their response. Nigeria was ranked lower than other countries such as South Africa, Togo and Kenya during the latest JEE in the linkage between public health and security authorities because Nigeria needed to have existing signed regulations and MOUs between security authorities and public health.^26^

#### Biosafety and biosecurity

Nigeria lacks proper, multi-sectorial biosecurity policies and programmes. Biosafety and biosecurity measures, along with plans for incident and emergency response, are essential for addressing any situations involving hazardous pathogens. In addition, Nigeria needs more Institutional Biosecurity programmes and national coordination of biosecurity activities. There are no consolidated institutions and locations with dangerous pathogens and toxin control. Lastly, laboratory audits are not utilised to assess the locations and present inventories of hazardous pathogens.^27^

#### Other weaknesses

The logistical complexities of expeditiously conveying resources and personnel to distant regions can pose significant obstacles in orchestrating a cohesive and synchronised reaction. Furthermore, the presence of obsolete disease monitoring equipment and disparities in health data accessibility among different states can also impede the analysis process.^28^

Moreover, a deficiency of confidence in the government may result in resistance to public health measures in specific communities. Additionally, the recurrent attrition of personnel and the progressive exit of skilled professionals responsible for managing outbreaks can ultimately diminish an organisation’s accumulated knowledge and proficiency. In certain situations, there may be a persistence of administrative inefficiencies and a need for coordination between local and central authorities.^28^

### Recommendations

The following are recommended to strengthen Nigeria’s public health response to infectious diseases:

- *Public health infrastructure*: Increase investment in healthcare infrastructure, including hospitals, clinics and laboratories. Train and recruit more healthcare workers who adequately respond to infectious disease outbreaks.
- *Funding*: Currently, there is a shortage of funding to prevent pandemic diseases in Nigeria. Therefore, securing external and internal funds for outbreak preparedness and response is imperative.^28^
- *Address poverty and inequality*: Implement policies and programmes to reduce poverty and inequality. This will also improve access to healthcare and other essential services for all Nigerians.
- *Increase efforts at the subnational level*: This can be done by establishing Public Health Emergency Operations Centres (PHEOCs) at the local government levels. This will improve coordination and collaboration at the subnational level, which is crucial for effective outbreak management.^29^ Additionally, this includes increases in funding by 50%, setting a public health security legal framework and ensuring effective advocacy.^30^

## Conclusion

In the past two decades, Nigeria has struggled with approximately six infectious diseases of significant public health concern. Among these are COVID-19, LF, Ebola virus, meningitis, diphtheria and cholera. These diseases have been designated as public health priorities because of their high incidence rates or high infectivity, such as COVID-19 and diphtheria, as well as by their low incidence rates but high case fatality rates, as seen in zoonotic haemorrhagic fever diseases such as LF and Ebola virus.

In response, the nation has enhanced its infectious disease preparedness and response capacities in recent years, as evidenced by the results of the 2023 JEE, which showed significant improvements. The areas of improvement include surveillance, workforce development and prevention at entry points. Furthermore, strong risk communication and community engagement, workforce development and establishment of good national reference libraries for confirmation of suspected cases are also included.

However, despite its strengths, persistent public health response weaknesses impede optimal outbreak containment. These include inadequate funding, poor efforts at the subnational level and poor linkage between public health and security authorities. In addition, there is a lack of integration of the animal sector, biosafety and biosecurity policies and programmes and logistical complexities. Therefore, it is necessary to invest in disease surveillance, the training of the healthcare workforce, diagnostic technologies and emergency response platforms, which will be crucial in strengthening Nigeria’s capacity to control future outbreaks. By investing in these areas, Nigeria can enhance its ability to detect, respond to and contain outbreaks swiftly and effectively. In addition to these investments, Nigeria can further improve its outbreak response by addressing poverty and inequalities, increasing efforts at the subnational levels through effective community engagement and establishing a Memorandum of Understanding (MOU) involving the public health sector and law enforcement agencies.
